# Immunodominance is a poor predictor of vaccine-induced T follicular helper cell quality

**DOI:** 10.1016/j.ebiom.2026.106185

**Published:** 2026-02-26

**Authors:** Ming Z.M. Zheng, Hyon-Xhi Tan, Kathleen M. Wragg, Lydia Murdiyarso, Devaki Pilapitiya, Andrew Kelly, Robyn Esterbauer, Christopher A. Gonelli, Adam K. Wheatley, Jennifer A. Juno

**Affiliations:** Department of Microbiology and Immunology, The University of Melbourne at the Peter Doherty Institute for Infection and Immunity, Melbourne, Victoria, 3000, Australia

**Keywords:** Vaccine, T follicular helper cell, CD4 T cell, Germinal centre, Immunodominance

## Abstract

**Background:**

Rational engineering of vaccine immunogens to focus B cell responses on potently neutralising epitopes is a promising approach to improve the potency, breadth and durability of viral vaccines. Such strategies, however, can compromise vaccine immunogenicity through the unintended exclusion of CD4+ T cell epitopes, which are critical for the development of T follicular helper (TFH) cells and to support high affinity antibody production.

**Methods:**

Using a prototypic influenza haemagglutinin (HA) stem immunogen lacking effective CD4+ T cell help in C57BL/6 mice, we interrogated the minimal requirements for T cell help needed to drive serological responses to vaccination.

**Findings:**

We find that priming of naïve CD4+ T cells is markedly efficient, however the immunodominance of a given CD4+ T cell epitope is not predictive of the propensity to provide high quality help to antigen-specific B cells. In the context of soluble antigens, provision of a single MHC class II epitope is sufficient to drive robust germinal centre responses and serum IgG titres. However not all CD4+ epitopes provide equivalent levels of B cell help, despite priming comparable numbers of antigen-specific CD4+ T cells. Finally, we show multimerizing and arraying antigens on nanoparticle scaffolds unlocks highly subdominant, near-undetectable CD4+ T cell helper responses to support a T-dependent antibody response.

**Interpretation:**

Our findings emphasise the importance of CD4+ T cell help for programming robust and durable humoural immunity, and provide crucial insights to guide the rational incorporation of favourable T cell epitopes into vaccines.

**Funding:**

The study was funded by the 10.13039/501100000925NHMRC.

**Research in context**

**Evidence before this study**

The requirement for effective CD4 T cell help in initiation and maintenance of the germinal centre (GC) reaction is well established. Studies have demonstrated that poorly immunogenic vaccine antigens can be supplemented with additional T cell help via linkage to carrier proteins or nanoparticle scaffolds, thereby improving antibody production. What is unclear, however, is the extent to which the epitope specificity and immunodominance of polyclonal T cell populations impact the quality of help provided to antigen-specific B cells.

**Added value of this study**

This study demonstrates that polyclonal CD4 T cell populations vary substantially in their ability to support GC reactions. The intrinsic ‘helpfulness’ of a given polyclonal T cell population is unrelated to immunodominance, and is most closely tied to the ability to transition to T follicular helper cells that actually reside within the GC. In some cases, a single epitope is sufficient to robustly reverse the immunological silence of an immunogen such as the influenza HA stem. Less immunocapable epitopes can provide B cell help in a highly context dependent manner, and may require antigen multimerization or concerted B cell/T cell interactions to effectively support GC formation and immunogenicity.

**Implications of all the available evidence**

Collectively, these data underscore a qualitative variability in B cell help provided by distinct epitope-specific CD4 T cells. Given that quality is not predicted by immunodominance, identification of optimal epitopes for inclusion in vaccine antigens will require experimental validation. Nonetheless, the ability of individual epitopes to drive potent GC responses provides a tractable avenue to rationally improve vaccine immunogenicity.

## Introduction

The development of effective vaccines has fundamentally reshaped the ability of human populations to contain and control infectious diseases. While historically vaccines were derived from attenuated or killed whole pathogens, more recent vaccine development efforts have focused upon identification of the most protective “subunits” for inclusion as vaccine immunogens. This was extended during the COVID-19 pandemic, where rational vaccine design was used to engineer the SARS-CoV-2 spike immunogens in protein, mRNA or viral-based vaccines to structurally stabilise^1^^,^^2^ and/or prevent cleavage^3^^,^^4^ of the spike trimer. Analogous modifications of the F protein are incorporated into recently approved vaccines for Respiratory Syncytial Virus (RSV).^5^ Further efforts to concentrate immunity upon conserved or potently neutralising domains have seen re-engineering of vaccine immunogens to the level of isolated protein subdomains such as the SARS-CoV-2 receptor-binding domain (RBD)^6^^,^^7^ or stem region of influenza haemagglutinin (HA).^8^ While such small protein targets effectively focus immune recognition by B cells, increasing evidence suggests this might come at a cost to immunogenicity, seen in both pre-clinical models^9^^,^^10^ and human clinical trials.^11^^,^^12^

Outside of the stochasticity of vaccine delivery, the immunogenicity of vaccine antigens varies, likely reflecting a combination of protein intrinsic factors and host genetics. We and others have identified that a paucity of CD4+ helper T cell epitopes is one important factor constraining vaccine immunogenicity.^9^^,^^10^^,^^13^^,^^14^ Unlike B cells, whose immunoglobulin receptors scan and engage the near infinite diversity of conformational epitopes on protein surfaces, CD4+ T cells can only recognise linear peptide epitopes in the context of a host MHC-II. This heightened restriction limits the absolute number of T cell epitopes within any given immunogen, with distribution being uneven and individualistic.^15^ Reducing the size of a vaccine immunogen therefore has the potential to concomitantly shrink the pool of available CD4+ T cell epitopes, leaving some antigens poorly recognised even in diverse human populations.^16^, ^17^, ^18^ In extreme examples, exemplified by model HA stem^9^ or hen egg lysozyme (HEL)^19^ immunogens in genetically inbred C57BL/6 (BL/6) mice, a lack of CD4+ T cell epitopes can render immunisation effectively immunologically silent.

Various strategies have been reported to augment CD4+ T cell priming, T follicular helper cell (TFH) differentiation, and subsequent vaccine immunogenicity, but generalised applicability, comparative utility, and mechanisms of action remain unclear. Presentation of vaccine antigens on nanoparticle scaffolds enhances B cell receptor (BCR) recognition and signalling,^20^ but can also augment CD4+ T cell help via epitopes localised within the scaffold, as demonstrated for ferritin nanoparticle vaccines.^21^ Similarly, covalent coupling of poorly immunogenic proteins to carrier proteins or alternate sources of T cell help can supplement the available antigen-specific CD4+ T cell pool,^9^^,^^22^ analogous to the carrier proteins essential for driving antibody responses to pneumococcal carbohydrates in childhood vaccines.^23^ However the impacts of epitope specificity or relative immunodominance of polyclonal CD4+ T cell populations upon the capacity to provide help to germinal centre (GC) B cells is poorly understood.

Here, we made use of the HA stem-C57BL/6 model^9^ to address the features of CD4+ T cell immunity that drive potent humoural immune responses to vaccination. We find evidence for a spectrum of epitope-level regulation of GC initiation, with some CD4+ T cell specificities unable to support antigen-specific B cell proliferation and IgG production. Critically, epitope immunodominance did not necessarily predict the quality of help provided by antigen-specific CD4+ T cells: some immunodominant epitopes exhibited no helper capability, while subdominant responses provided effective help, with even CD4+ T cells at near-undetectable levels successfully contributing to GC formation in the context of multimerized antigen. Overall, these data demonstrate that while vaccine immunogenicity is critically dependent on CD4+ T cell availability, the capacity to support humoural responses is not universal to all CD4+ T cell epitopes, with implications for the rational design of small, engineered vaccine immunogens.

## Methods

### Mouse ethics, immunisations and infections

C57BL/6, BALB/c, and SMARTA-transgenic (C57BL/6 background) mice were bred in-house under specific pathogen-free conditions in the animal facility at the Peter Doherty Institute of Infection and Immunity, University of Melbourne, Australia. Mouse studies were carried out in accordance with the University of Melbourne Animal Ethics Committee (approval no. 22954). All mice were female and aged 6–12 weeks at the time experiments commenced. A total of 5 μg (unless otherwise indicated) of protein, peptide, or nanoparticle were formulated in phosphate buffered saline (PBS) at a 1:1 ratio with Addavax (InvivoGen, cat#INV-vac-adx-10) adjuvant in a total volume of 100 μL. Mice were anaesthetised by isoflurane inhalation with oxygen flow at 2 L/min and isoflurane vapouriser set to 3 (Stinger Anaesthetic Machine), prior to 50 μl intramuscular injections at the left and right quadriceps using a 29G needle. For influenza infections, mice were anaesthetised as above and intranasally infected in a volume of 50 μl with a sublethal dose of 50 TCID_50_ of A/Puerto Rico/8/34 (PR8). At experimental endpoints, mice were killed using CO_2_ asphyxiation using a delivery of 50% chamber volume per minute.

### Protein expression

Stem and Stem-Ferritin immunogens were prepared in-house, as previously described.^9^^,^^24^ Briefly, stabilised HA stem proteins were engineered for A/Puerto Rico/08/1934 using methods established previously for the design of Gen6 HA stem in Yassine et al.^25^ Stem-Ferritin nanoparticles were expressed by transient transfection of Expi293F (Life Technologies, Thermo Fisher Scientific) suspension cultures and purified using ion exchange chromatography with HiTrap Q HP column (GE Healthcare) and exclusion chromatography.

For stem proteins conjugated to CD4+ T cell epitopes, expression constructs substituting the original Avitag for peptide sequences for OTII (ISQAVHAAHAEINEAG), HA_91_ (RSWSYIVETPNSENGIC), HA_115_ (YEELREQLSSVSSFERF), HA_301_ (AINSSLPYQNIHPVTIG), HA_523_ (SMGIYQILAIYSTVASS) and GP_61_ (GLKGPDIYKGVYQFKSVEFD) were synthesised (GeneArt), cloned into mammalian expression vectors and expressed via transient transfection of Expi293 suspension cultures (Life Technologies, Thermo Fisher Scientific). Proteins were purified by polyhistadine-tag affinity chromatography and gel filtration.

### Enzyme-linked immunosorbent assay (ELISA)

Blood samples for serum isolation were collected either by submandibular bleed or terminal cardiac puncture bleed using a 26G needle. 96-well MaxiSorp plates (ThermoFisher, cat# 3442404) were coated with 2 μg/mL recombinant stem or OVA protein overnight at 4 °C. Plates were washed with 0.05% (v/v) Tween 20 (Sigma, cat# P1379) in PBS and blocked with 1% (v/v) FCS in PBS for 1 h, room temperature, before incubation with serial dilutions of sera for 2 h. Plates were washed and horseradish peroxidase-conjugated anti-mouse IgG (1:15,000; Seracare, cat# 5450-0011) was added for 1 h. After washing, plates were developed with 3,3′,5,5′-Tetramethylbenzidine (TMB; ThermoFisher, cat#SB02), stopped with 0.16 M sulphuric acid (Sigma, cat# 84727) and the absorbance measured at 450 nm on FLUOstar Omega microplate reader (BMG Labtech). Curves were fitted (four-parameter log regression) and end-point titrations calculated as the reciprocal serum dilution yielding 2x background using GraphPad Prism version 10.

### Generation of B cell probes and peptide MHC II tetramers

Recombinant stem protein was biotinylated using BirA (Avidity) and stored at −80 °C. Conjugation was performed by sequential addition of streptavidin-PE or -APC (Life Technologies, cat#S866 and S868) or streptavidin-BV711 (BD, cat# 563262). Mouse H2-IAb RSWSYIVETPNSENGI PE-conjugated tetramer (IAb/HA_91_) was generated by ProImmune. Biotinylated mouse H2-IAb DIYKGVYQFKSV monomer (IAb/GP_61_; ProImmune) was tetramerized by sequential addition of streptavidin-PE (Life Technologies, cat# S866).

### Detection of antigen-specific B and T cells ex vivo

Iliac and inguinal vaccine-draining lymph nodes were passed through a 70 μm filter, centrifuged (500 *g*, 7 min) and washed with PBS prior to viability staining with live/dead red (Invitrogen, cat# L34972) for 3 min. Cells were Fc blocked with anti-CD16/32 antibody (BioLegend, cat# 101302) for 10 min and stained with surface antibodies of interest (Table 1) for 30 min at 4 °C. Cells were then washed with 2% (v/v) FCS in PBS and fixed with 1% (v/v) formaldehyde (BD, cat# 554655) before acquisition on a BD Symphony or Fortessa flow cytometer. For detection of antigen-specific B cells, PE, APC or BV711 conjugated stem probes were included in the surface staining antibody cocktail. For detection of antigen-specific T cells, single-cell suspensions were pre-incubated at 37 °C with 50 nM dasatinib (Sigma, cat# SML2589) in 2% (v/v) FCS in PBS containing 1 μg/mL anti-TCRβ (BD, cat# 553167). After 30 min, 4 μg/mL of IAb/HA_91_ or IAb/GP_61_ tetramers were added for a further 3 h, before proceeding with viability and surface staining. Data were analysed using FlowJo (v10.10, BD Biosciences). Data collection and analysis were not performed blind to the conditions of the experiments.

**Table 1:** List of antibodies for flow cytometry analysis.

Marker	Fluoro-chrome	Clone	Supplier	Catalogue number	Dilution	Panel	RRID
CD154	BV650	MR-1	BD	740,480	1:250	AIM	AB_2740205
OX-40	PE Cy7	OX-86	BioLegend	119,416	1:100	AIM	AB_2566155
PD-1	BV786	29F.1A12	BioLegend	135,225	1:100	AIM	AB_2563680
CD4	BUV737	RM4-5	BD	612,843	1:200	AIM, proliferation	AB_2870165
CD3	APC Fire 750	145-2C11	BioLegend	100,362	1:50	AIM, proliferation	AB_2629687
B220	BV605	RA3-6B2	BD	563,708	1:100	AIM, proliferation	AB_2738383
CD25	BB515	PC61	BD	564,424	1:50	AIM, Combined B + T	AB_2738803
CD44	BB700	IM7	BD	566,506	1:50, 1:1000	AIM, Combined B + T	AB_2744396
F4/80	PE Dazzle	BM8	BioLegend	123,146	1:100, 1:200	AIM, Combined B + T	AB_2564133
CXCR5	BV421	L138D7	BioLegend	145,512	1:50, 3:200	AIM, Combined B + T	AB_2562128
IgD	BUV395	11-26c.2a	BD	564,274	1:143	Combined B + T	AB_2738723
CD3	BUV737	145-2C11	BD	612,771	1:100	Combined B + T	AB_2870100
B220	BUV805	RA3-6B2	BD	569,199	1:143	Combined B + T	AB_3684861
CD45.2	V500	104	BD	562,129	3:200	Combined B + T	AB_10897142
CD138	BV605	281-2	BioLegend	142,531	1:200	Combined B + T	AB_2715767
CD4	BV650	RM4-5	BD	563,747	1:333	Combined B + T	AB_2716859
GL7	Alexa 488	GL7	BioLegend	144,612	7:20,000	Combined B + T	AB_2563285
CD98	PE Cy7	4F2	BioLegend	128,213	1:1000	Combined B + T	AB_2750546
CD38	APC Fire 750	90	BioLegend	102,738	1:200	Combined B + T	AB_2876402
CD90.2	BUV496	53-2.1	BD	741,046	1:200	Combined B + T	AB_2870661
CD62L	BUV395	MEL-14	BD	569,400	1:200	Combined B + T	AB_3685037

### Activation induced marker assays

Individual HA, stem (BEI Resources) or ferritin (GenScript) peptides (15-mer with 11 amino acid overlap) were pooled and used to detect antigen-specific CD4+ T cell responses *in vitro*. LN single cell suspensions were cultured in 96-well round-bottom plates in RPMI-1640 (Thermo Fisher Scientific) with 10% (v/v) FCS and 2% (v/v) penicillin-streptomycin (RF10) media containing 20 mM A438079 (Santa Cruz, cat# 203788), anti-mouse CD154 antibody and peptide pool of interest (2 mg/mL/peptide) or an equivalent volume of DMSO (Sigma, cat# D1435). Following 18hr of stimulation, cells were washed in PBS, stained for viability (3 min at room temperature) and then stained with surface antibodies of interest (Table 1).

### T cell proliferation assay

To generate single-cell suspensions, spleens were passed through a 70 μm filter and centrifuged (500×*g*, 7 min). Red blood cells were lysed using 1x BD PharmLyse (Cat #555899) for 3 min, and the reaction quenched with 1x PBS. Splenic single-cell suspensions were labelled with 2.5 mM of CellTrace Violet (CTV; Invitrogen, cat#C34557) for 10 min and washed twice (500×*g*, 7 min) with RF10. 4 × 10^5^ CTV-labelled cells were cultured in 96-well round-bottom plates in RF10 with 0.5 mg/mL HA, stem or ferritin peptide pools, or anti-CD3/CD28 Dynabeads (Gibco, cat # 11456D) for 4 days at 37 °C, prior to staining for flow cytometric analysis (Table 1).

### Immunofluorescent microscopy

Fresh tissues were snap-frozen in Tissue-Tek O.C.T. compound (Sakura Finetek USA) and stored at −80 °C. 7 μm sections were cut using the Leica CM3050S cryostat (Leica Biosystems). Prior to staining, sectioned tissues were fixed in cold acetone solution (Sigma) for 10 min, rehydrated with PBS for 10 min, and then blocked in 5% (w/v) bovine serum albumin (Sigma) and 2% (v/v) normal goat serum (Jackson ImmunoResearch). A cocktail of antibodies including GL7 AF488 (clone GL7, BioLegend) and B220 BV421 (clone RA3-6B2, BioLegend) were added for 1 h at RT. Slides were mounted with ProLong Diamond Antifade Mountant (Life Technologies). Tiled z-stack images at 20x magnification (0.8 numerical aperture) and 1 airy unit were acquired on a LSM780 microscope (ZEISS) and analysed with Fiji software.^26^

### Statistical analysis

Data are presented as median ± interquartile range. All statistical analysis was performed in Prism 10 (GraphPad) using nonparametric statistical tests as indicated (making no assumptions about data normality). P < 0.05 was considered statistically significant.

### Role of funders

The funders had no role in the study design, data collection, data analyses, interpretation, or writing of report.

## Results

### Multimeric display of HA stem antigens on self-assembling ferritin nanoparticles compensates for highly subdominant CD4 help

**Fig. 1:**
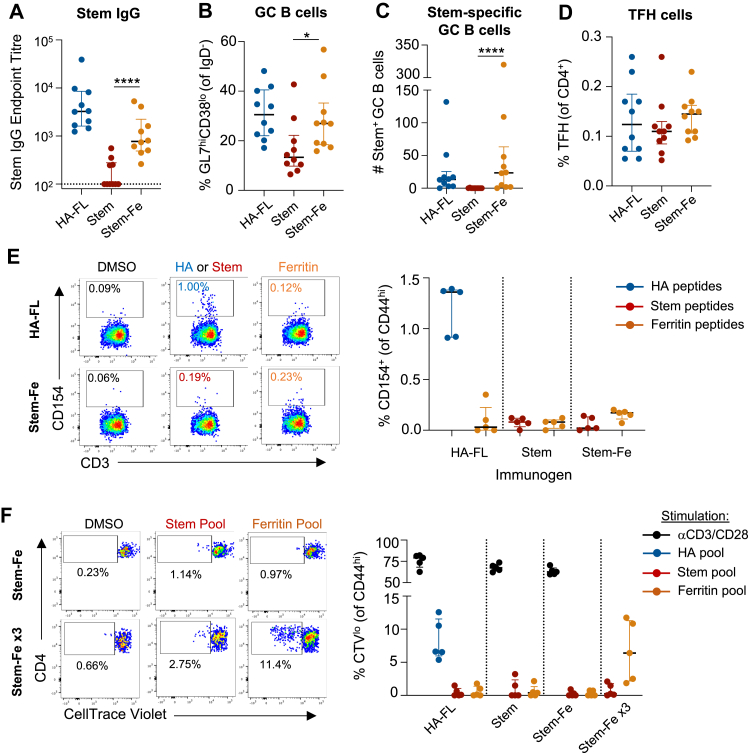
**Nanoparticle display enhances stem immunogenicity in the absence of prominent CD4^+^ T cell help.** Mice were vaccinated with 5 mg of full-length HA protein (HA-FL), HA-stem protein, or stem-ferritin nanoparticles (Stem-Fe) co-formulated 1:1 with Addavax adjuvant. Serum and draining LN were collected at day 14 post-vaccination. **(A)** Serum endpoint titres of stem-specific IgG (N = 10/group). **(B)** Frequency of GL7^hi^CD38^lo^ GC B cells, **(C)** number of stem-specific GC B cells, or **(D)** frequency of CXCR5^hi^PD-1^hi^ TFH in the draining LN (N = 10/group). **(E)** Representative staining and frequency of CD154^+^ stem or HA-specific memory CD4^+^ T cells following *in vitro* stimulation. Frequencies are background subtracted based on the DMSO control (N = 5/group). **(F)** CD4^+^ T cell proliferation following *in vitro* peptide stimulation with HA, stem or ferritin peptide pools. Splenocytes were harvested from vaccinated mice at day 14, or at day 79 after 3 vaccinations (N = 5/group). Lines indicate median and IQR. Statistics assessed by Mann–Whitney test comparing Stem and Stem-Fe groups. ∗p < 0.05, ∗∗∗∗p = 0.0001.

We previously established primary vaccination of BL/6 mice with a soluble HA stem protein fails to elicit an antigen-specific GC B cell response or production of serum IgG due to a lack of available CD4+ T cell epitopes.^9^ Presentation of stem on self-assembling ferritin nanoparticles enhances its immunogenicity^25^^,^^27^ but the source of T cell help in this system is unclear, as BL/6 mice are reported to lack ferritin-specific CD4+ T cells.^21^ We find that stem-ferritin nanoparticles (Stem-Fe) are robustly immunogenic, eliciting significantly higher stem IgG titres than soluble stem trimers (p = 0.0001; Fig. 1A) and greater numbers of bulk and stem-specific GC B cells (p = 0.02 and p = 0.0001, respectively; Fig. 1B and C, gating in Supplementary Fig S1). Using confocal imaging and flow cytometry, we confirmed the GL7^hi^CD38^lo^ B cell populations in stem-Fe vaccinated mice reflected *bona fide* GC structures. GL7 expression was correctly localised to GC-like structures within the follicle (Supplementary Fig S2A), and canonical light zone and dark zone GC B cell populations were observed in both full-length HA (HA-FL) and stem-Fe animals (Supplementary Fig S2B), indicative of a T-dependent GC reaction.

**Fig. 2:**
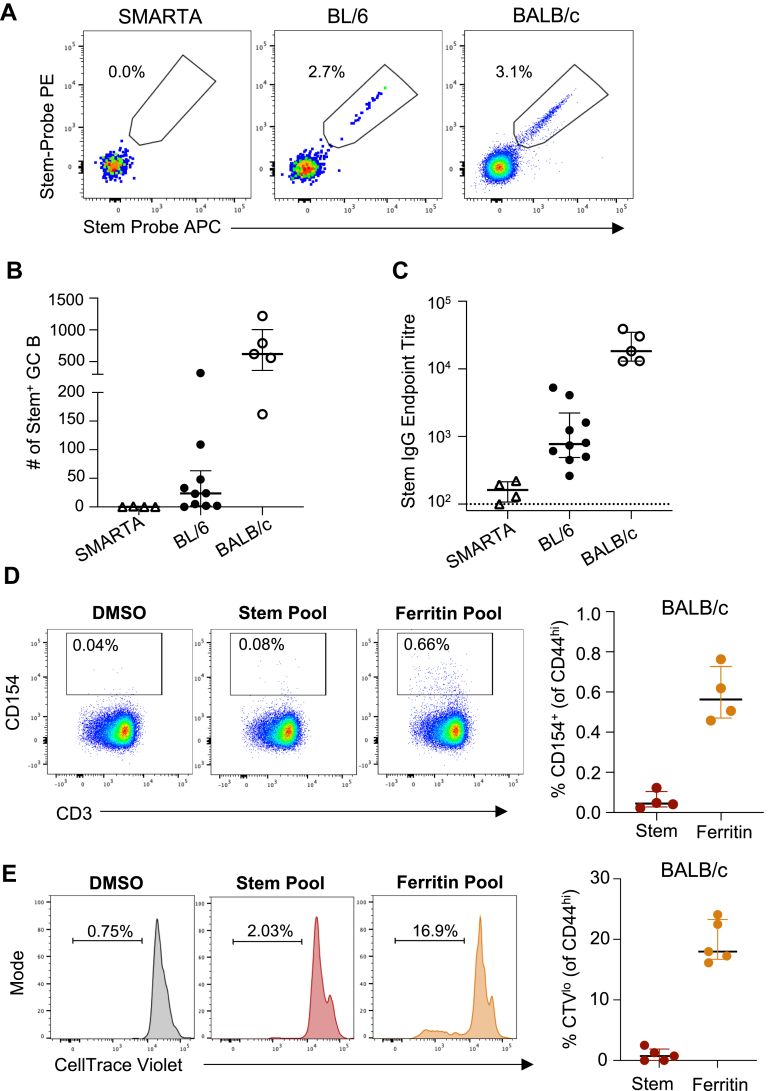
**Stem-Fe nanoparticle immunogenicity in SMARTA and BALB/c mice.** Stem-specific **(A)** GC B cell frequencies, **(B)** GC B cell counts or **(C)** IgG titres at day 14 following Stem-Fe vaccination of SMARTA (N = 4), WT BL6 (N = 10) or BALB/c (N = 5) mice. **(D)** Stem- or ferritin-specific CD4^+^ T cell responses in the draining LN of Stem-Fe vaccinated BALB/c mice at day 14 (n = 4). **(E)** CD4^+^ T cell proliferation in BALB/c mice following *in vitro* peptide stimulation with DMSO, stem or ferritin peptide pools. Splenocytes were harvested from vaccinated mice at day 14 (N = 5/group). Lines indicate median and IQR.

Given the lack of identifiable CD4+ T cell epitopes within the stem antigen and the limited impact of Stem-Fe vaccination on total TFH frequency or number (Fig. 1D, Supplementary Fig S2C), we assessed the magnitude of ferritin-specific responses. Consistent with previous reports for both stem^9^ and ferritin^21^ immunogens, we observed limited CD4+ T cell responsiveness upon *in vitro* re-stimulation with either peptide pool (Fig. 1E). Similarly, use of a proliferation assay for sensitive detection of any low-frequency populations of stem- or ferritin-specific T cells revealed only sporadic, low-level proliferation when compared to the vehicle control (Fig. 1F). To further amplify antigen-specific T cell frequencies, we primed and then boosted mice twice on days 21 and 42 with Stem-Fe. Three out of five Stem-Fe vaccinated animals exhibited clear proliferative responses to the ferritin peptide pool (Fig. 2F), albeit with only 3–4 cell divisions over the course of the 4-day stimulation. Collectively, these data suggest highly subdominant epitopes within ferritin could be the source of T cell help supporting Stem-Fe immunogenicity.

If ferritin-specific CD4+ help below the detection limit of conventional T cell assays was responsible for supporting a primary GC reaction, we reasoned that the Stem-Fe antigen should not be immunogenic in transgenic (tg) BL/6 mice that express a single T cell receptor (TCR). Conversely, animals with high frequencies of ferritin-specific T cells (i.e. BALB/c mice as previously demonstrated^21^) should produce superior serological responses. Stem-Fe vaccination of SMARTA tg (BL/6 background), wild-type (WT) BL/6 and WT BALB/c animals demonstrated a striking immunogenicity gradient, with negligible numbers of stem-specific GC B cells (Fig. 2A and B) or IgG (Fig. 2C) detected in SMARTA mice, while responses in WT BALB/c were more than 20-fold higher compared to WT BL/6 (Fig. 2A–C). We confirmed that ferritin-specific CD4+ T cells were readily detectible in BALB/c mice by both standard restimulation (Fig. 2D) and proliferation assays (Fig. 2E), demonstrating that the ferritin core provides substantially more CD4+ T cell help in BALB/c versus BL/6 mice.

Overall, when stem is arrayed on the surface of a nanoparticle, the low level of ferritin-specific CD4+ T cell help available in BL/6 mice becomes sufficient to support a stem-specific GC response that is otherwise absent following soluble stem vaccination.

### The TFH repertoire of full-length HA is limited in breadth and dominated by a single epitope

The gradient of Stem-Fe immunogenicity across SMARTA, BL/6 and BALB/c mice strains suggests that availability of CD4+ T cell help acts as a rheostat that directly tunes the magnitude of the GC and resultant serological response. While we have established the near-complete lack of CD4+ T cell help in stem, the breadth and specificity of epitopes that successfully support the immunogenicity of the HA-FL protein requires detailed mapping.

**Fig. 3:**
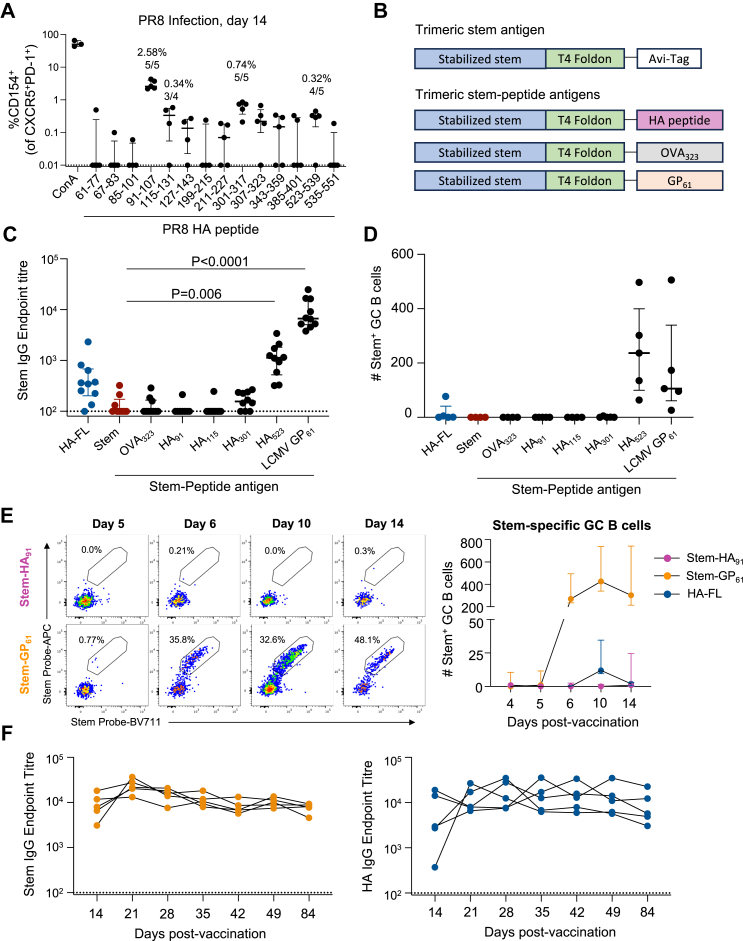
**Rescue of stem immunogenicity by genetically fused CD4^+^ T cell epitopes. (A)** Identification of immunogenic peptides following intranasal infection with PR8 virus. Mediastinal LN were collected on day 14 and restimulated *in vitro* with DMSO, ConA or indicated peptides (N = 4–5 mice per peptide). Immunogenic peptides are labelled with the median response and number of responding mice. **(B)** Design of trimeric stem antigens covalently linked to HA-derived peptides or prototypic OVA_323_ and GP_61_ peptides. **(C)** Stem-specific IgG endpoint titres at day 14 post-vaccination with 5 μg of HA-FL, stem, or stem-peptide antigens formulated with Addavax (N = 7–10 per group). Statistics assessed by Kruskal–Wallis test with Dunn's post-test compared to the stem control. **(D)** Number of stem-specific GC B cells (GL7^+^CD38^lo^) at day 14 post-vaccination (N = 4–5 per group). **(E)** Longitudinal tracking of stem-specific GC B cells in the draining LN at days 4, 5, 6, 10 and 14 post-vaccination (N = 5 per group). **(F)** Durability of antigen-specific serum IgG following stem-GP_61_ or HA-FL vaccination (N = 5 per group). Symbols or lines indicate median with IQR.

To maximise the number of TFH for screening, BL/6 mice were infected with a sublethal dose of PR8 influenza virus and mediastinal LN (mLN) which drain the site of infection were collected on day 14. Pooled LN cell suspensions were stimulated *in vitro* with a matrix of 20 peptide pools spanning the HA protein (Supplementary Fig S3A), with candidate immunogenic peptides selected by identifying pools that elicited a CD154 response greater than the DMSO control (Supplementary Fig S3B). Individual peptide screening identified 14 potential hits, which we subsequently tested for consistent recognition across multiple animals. Nine peptides elicited CD4+ T cell responses in at least 3 of 5 mice, with 4 putative epitopes identified for the CXCR5^+^PD-1^+^ CD4+ T cell population (enriched for pre-TFH/TFH cells; Fig. 3A). HA_91–107_ was identified as highly immunodominant, with a median of 2.6% of CXCR5^+^ cells specific for this single peptide. Three other epitopes elicited lower magnitude responses: HA_301–323_ (two overlapping peptides spanning HA_301–317_ and HA_307–323_), HA_115–131_, and HA_523–539_ (Fig. 3A). A similar hierarchy was observed within the total CD4+ T cell population (Supplementary Fig S3C), with no immunogenic peptides located within the HA-stem domain as expected.

To confirm whether HA_91_ was similarly immunodominant in the context of vaccination, we intramuscularly immunised mice with 5 μg of soluble HA-FL protein in Addavax adjuvant. Here, the HA_91_ peptide accounted for 74% of the CD154 response to the full HA peptide pool (median of 7.85% CD154^+^ for HA pool versus 5.76% for HA_91_ peptide) (Supplementary Fig S3D).

### Addition of an MHC class II epitope to soluble stem protein rescues stem-specific IgG

Given the wide spectrum of TFH responses capable of modulating serological outcomes, from low-level ferritin-specific help for nanoparticle-driven GCs to the heavy dominance of HA_91_ in the native HA-specific T cell pool, we further sought to clarify the intrinsic “helpfulness” of discrete CD4+ T cell epitopes. We tested whether incorporation of a single epitope onto the stem vaccine antigen could prime cognate CD4+ T cells *in vivo* and thereby improve the stem-specific IgG response. Variants of the trimeric stem immunogen were developed with addition of a single peptide epitope sequence adjoined at the C-terminus of the T4 foldon domain (Fig. 3B). We produced immunogens incorporating each peptide mapped from HA (HA_91_, HA_115_, HA_301_ and HA_523_) as well as prototypic IA^b^-restricted peptides from ovalbumin (OVA_323_; ‘OTII’) and Lymphocytic Choriomeningitis Virus (LCMV) glycoprotein (GP_61_; ‘SMARTA’).

WT BL/6 mice were vaccinated with a single 5 μg dose of HA-FL, stem, or stem variant antigens formulated in Addavax. On day 14, stem IgG titres were assessed by ELISA and stem-specific GC B cells quantified in the draining lymph node (dLN). Only two antigens, stem-HA_523_ and stem-GP_61_, were capable of eliciting stem-specific IgG and GC B cells at levels above the stem control (p = 0.006 and p < 0.0001, respectively; Fig. 3C and D). The failure of the immunodominant HA_91_ epitope to rescue the stem response prompted us to examine the biogenesis of the GC response from days 4–14 post-vaccination. Stem-HA_91_ vaccinated animals failed to form a stem-specific GC B cell population at any timepoint, while stem-GP_61_-immunised animals exhibited a robust stem-specific GC response evident from day 6 onwards (Fig. 3E, Supplementary Fig S4A). At peak, antigen-specific GC B cell numbers were 33-fold higher in Stem-GP_61_ vaccinated animals compared to HA-FL, with the serological response demonstrating similar durability for both antigens (Fig. 3F).

### HA_91_-specific TFH cell fail to populate the germinal centre

Despite HA_91_ dominance in the native CD4+ T cell response to HA-FL vaccination and PR8 infection, incorporation of the HA_91_ peptide failed to rescue stem immunogenicity. Possible mechanisms could include a peptide processing defect in the engineered stem immunogen that prevented or reduced naïve CD4+ T cell priming by DCs, or alternatively a qualitative defect in TFH differentiation and/or T cell:B cell interactions. To address this, we produced an IA^b^/HA_91_ tetramer that facilitated tracking of antigen-specific T cells. In both PR8 infection and HA vaccination, staining of the draining LN with tetramer confirmed the expansion of a prominent CD44^hi^ epitope-specific CD4+ T cell population (Supplementary Fig S4A). A similar tetramer successfully identified GP_61_-specific T cells in dLN following LCMV GP immunisation (Supplementary Fig S4B).

**Fig. 4:**
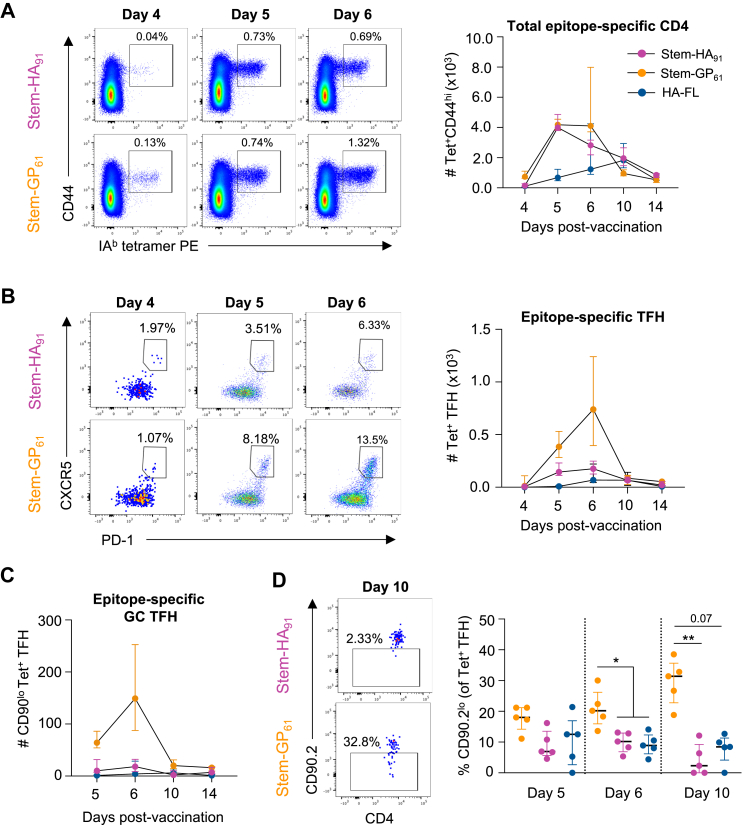
**Differential GC recruitment of HA_91_ and GP_61_-specific TFH populations. (A)** Longitudinal tracking of total, **(B)** TFH (CXCR5^hi^PD-1^hi^) or **(C)** GC resident TFH (CD90^lo^) antigen-specific CD4^+^ T cell numbers in the draining LN using IA^b^ tetramers. **(D)** Proportion of tetramer ^+^ TFH with CD90^lo^ phenotype at days 5, 6 and 10 post-vaccination. Plots show CD90 expression for the tetramer^+^ TFH gate. Symbols indicate median and IQR (N = 5 per group). Statistics assessed by Kruskal-Wallis test with Dunn's post-test. ∗p < 0.05, ∗∗p < 0.01.

Using the HA_91_ and GP_61_ tetramers, we compared epitope-specific CD4+ T cell numbers and phenotypes at days 4, 5, 6, 10 or 14 post-vaccination with either stem-HA_91_ or stem-GP_61_. Both antigens readily primed CD4+ T cells, evidenced by the expansion of a tetramer ^+^ CD44^hi^ population (Fig. 4A, Supplementary Fig S4C) with progressive maturation from a CD62L^hi^ to CD62L^lo^ phenotype (Supplementary Fig S4D). Total numbers of HA_91_-or GP_61_-specific T cells in the dLN were comparable, although the stem-HA_91_ antigen primed significantly more tetramer ^+^ cells at day 5 compared to HA-FL protein (Fig. 4A). Nevertheless, these populations followed different TFH differentiation trajectories over the subsequent 11 days. The small numbers of CXCR5^hi^PD-1^hi^ TFH-like GP_61_-specific cells seen on day 4 became a substantial TFH population by day 6, when median numbers of antigen-specific TFH were 4.2-fold greater for GP_61_ compared to HA_91_ (Fig. 4B, Supplementary Fig S4E).

Recent work has shown that the identification of TFH based on high CXCR5 and PD-1 expression includes cells located both within and adjacent to the GC.^28^ Downregulation of surface marker CD90 delineates a population of GC-resident TFH that emerge at day 5 post-immunisation and are dependent on MHCII-expressing B cells for their development and maintenance. Based on CD90 expression, we found significantly higher numbers of GC-resident TFH in stem-GP_61_ compared to stem-HA_91_ vaccinated animals from days 5–10 (Fig. 4C). While this was partially driven by the overall greater number of GP_61_-specific TFH, the dynamics of CD90 downregulation also differed between HA_91_ and GP_61_ TFH populations. From day 5–10, the proportion of GP_61_ TFH with a GC-resident phenotype increased from a median of 18.0%–31.4% (Fig. 4D, Supplementary Fig S4F). In contrast, the proportion of HA_91_ TFH recruited into the GC declined from 6.9% on day 5 to 2.3% on day 10 (Fig. 4D). As sustained GC B cell presentation of peptide:MHCII (pMHCII) is required to maintain CD90^lo^ TFH populations,^28^ these data suggested that suboptimal class-II presentation of the HA_91_ peptide by B cells may limit the development of GC-resident TFH.

### Broader B cell presentation of HA91 supports the Stem-HA91 GC response

**Fig. 5:**
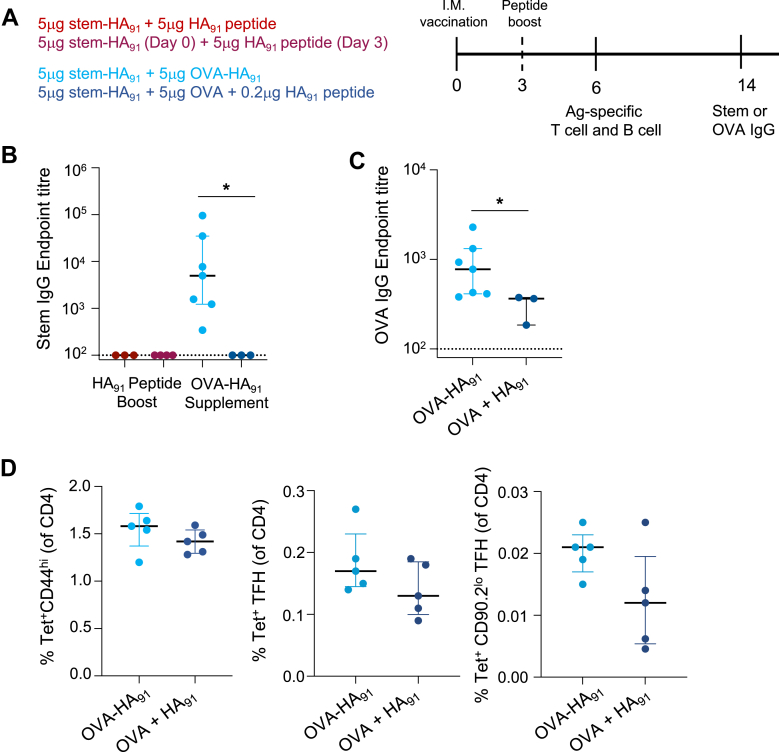
**Modulation of stem-HA_91_ immunogenicity through altered B cell antigen presentation. (A)** Overview of immunisation timeline and groups. (**B)** Stem or **(C)** OVA IgG titres at day 14 post-vaccination, related to the groups shown in panel A. N = 3–4 per group from either one or two independent experiments. Statistics assessed by Mann–Whitney test. **(D)** Frequencies of total, TFH, or GC TFH HA_91_Tet ^+^ T cells at day 6 post-vaccination with 5 μg stem-HA_91_ + 5 μg OVA-HA_91_ (light blue) or 5 μg stem-HA_91_ + 5 μg OVA + 0.2 μg HA_91_ (dark blue). N = 5 per group. Lines indicate median and IQR. ∗p < 0.05.

If poor peptide-MHCII presentation underpins the low immunogenicity of stem-HA_91_, then augmenting antigen presentation or promoting TFH differentiation through alternate pathways should rescue stem-HA_91_ immunogenicity (Fig. 5A). Prior studies have suggested that defects in TFH differentiation can be overcome when DC antigen presentation is extended by boosting a protein immunisation with cognate peptide three days later.^29^ However, vaccination with 5 μg of stem-HA_91_ supplemented with 5 μg of free HA_91_ peptide at either day 0 or day 3 failed to rescue stem antibody titres (Fig. 5B).

We next considered if the small pool of stem-specific naïve B cells may limit the presentation of pMHCII complexes, and contribute to the poor immunogenicity of stem-HA_91_ compared to HA-FL. We therefore immunised mice with stem-HA_91_ co-formulated with OVA-HA_91_ to increase the pool of naïve B cells presenting IA^b^/HA_91_, hypothesising that this could promote HA_91_-specific TFH differentiation and ultimately increase the quantity or quality of T cell help available for stem-specific B cells. As controls for the OVA-induced GC response and increased dose of HA_91_ peptide, mice were vaccinated with 5 μg of stem-HA_91_ co-formulated with 5 μg OVA and an equimolar amount (0.2 μg) of free HA_91_. Only animals vaccinated with stem-HA_91_ and OVA-HA_91_ exhibited stem-specific IgG at day 14 (Fig. 5B), suggesting that covalent linkage of HA_91_ to an additional B cell antigen was necessary for T cell-B cell interactions that supported a productive stem-specific GC. OVA-specific IgG titres were also significantly increased when animals were co-immunised with stem-HA_91_ and OVA-HA_91_ (Fig. 5C). Both groups exhibited similar HA_91_-specific T cell frequencies (Fig. 5D). While frequencies of bulk HA_91_ TFH were slightly higher in the stem-HA_91_+OVA-HA_91_ group (median 0.17% of CD4 versus 0.11% for stem-HA_91_+OVA + HA_91_), CD90^lo^ GC resident TFH were 1.75-fold more frequent (0.021% versus 0.012%) (Fig. 5D).

Collectively, these data suggest that the poor performance of the HA_91_ epitope as a sole source of CD4+ T cell help is at least partially attributable to a failure to establish or maintain an antigen-specific CD90^lo^ GC-resident TFH population, which can be mitigated by expanding the pool of B cells capable of presenting IA^b^/HA_91_ complexes.

### Antigen dose differentially impacts CD4+ T cell priming and TFH differentiation

**Fig. 6:**
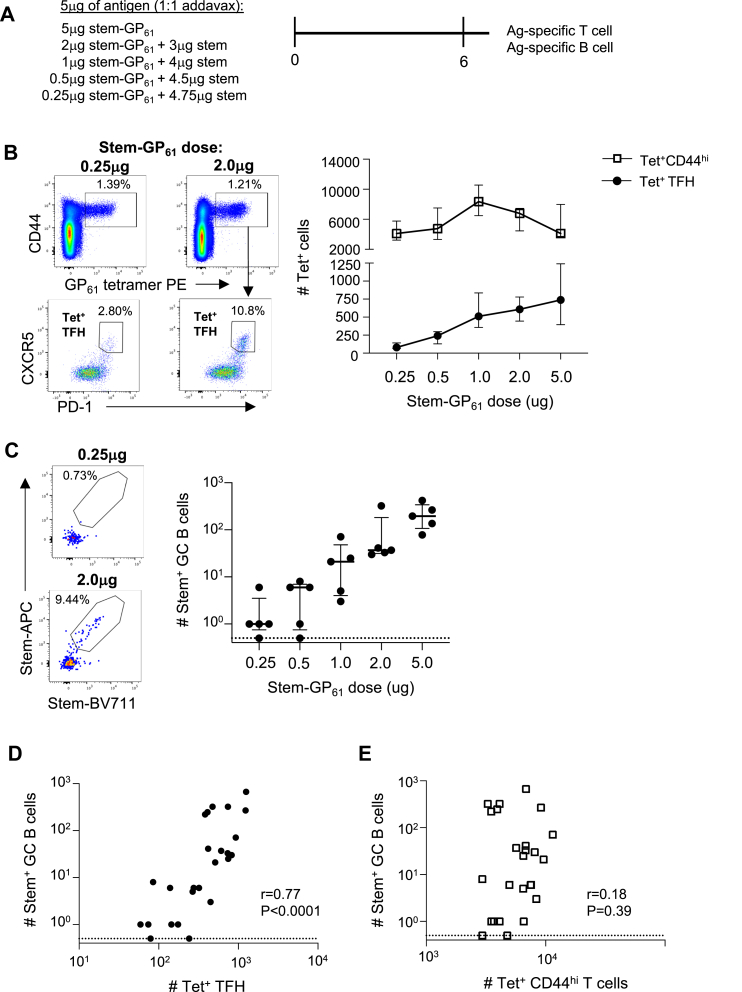
**Modulation of stem-**GP_61_**immunogenicity via altered B cell antigen presentation. (A)** Antigen doses for titration of GP_61_ peptide availability. **(B)** Numbers of total and TFH GP_61_ Tet ^+^ cells at day 6 post-vaccination (N = 5 per group). **(C)** Number of stem-specific GC B cells at day 6 post-vaccination (N = 5 per group). Symbols indicate median and IQR. **(D)** Correlation between number of Tet^+^ TFH or **(E)** total Tet^+^ cells with stem-specific GC B cells. Lymph nodes with no detectible stem-specific GC B cells were arbitrarily assigned a value of 0.5 (marked with a dashed line) for graphical display. Statistics assessed by Spearman correlation.

If differences in stem-HA_91_ and stem-GP_61_ immunogenicity were driven by pMHCII presentation in the GC, we next asked what the impact would be of reducing GP_61_ peptide availability. To titrate GP_61_ dose without changing the total amount of stem antigen available for B cell uptake, we vaccinated mice with varying doses of the stem-GP_61_ antigen supplemented with unmodified stem, such that the total dose of protein remained 5 μg (Fig. 6A). Draining lymph nodes were collected at day 6 post-vaccination, the timepoint of maximal GP_61_-specific T cell expansion and the appearance of stem-specific GC B cells. All immunisation doses elicited a similar number of total GP_61_-specific CD44^hi^ CD4+ T cells (Fig. 6B), highlighting the marked efficiency of T cell priming by DCs.

In contrast, we observed a dose-dependent increase in GP_61_-specific TFH differentiation (Fig. 6B) that parallelled a dose-dependent increase of stem-specific GC B cells (Fig. 6C). Accordingly, the number of stem + GC B cells correlated with the number of ag-specific TFH but not total numbers of ag-specific CD4+ T cells (Fig. 6D and E), reinforcing that when T cell priming and TFH differentiation are decoupled, GC formation is controlled by TFH availability. Collectively, these data suggest that vaccine dosing sufficient to establish efficient B cell presentation of pMHC to pre-TFH cells is markedly higher than doses required for DC-priming of naïve T cells, and that differences in B cell presentation of HA_91_ and GP_61_ peptides may underpin the differential immunogenicity of stem-HA_91_ and stem-GP_61_ antigens.

## Discussion

Engineering vaccine immunogens to reduce inclusion of B cell epitopes with weak or narrow protective capability is an attractive approach for maximising durable protection against challenging pathogens such as influenza, coronaviruses, Human Immunodeficiency Virus (HIV), and malaria. While such strategies facilitate highly tailored B cell engagement, increasing evidence suggests that a concomitant loss of CD4+ T cell epitopes can hinder overall vaccine immunogenicity *in vivo*. Here, we assessed how CD4+ T cell help supports productive GC reactions toward multimerized or soluble protein antigens, and found that T cell epitope specificity, rather than immunodominance, was a determining factor in TFH differentiation and vaccine immunogenicity.

Intrinsic protein immunogenicity is likely determined by the complex interplay of multiple factors including B cell precursor frequencies, antigen size, glycosylation, and the ability to elicit an effective CD4+ T helper cell response. In a polyclonal system with soluble viral antigens, gross immunogenicity is likely to reflect the totality of available CD4+ T cell help, with large antigens generally containing an increased number of CD4+ T cell epitopes than small antigens. This is consistent with the observed immunogenicity gradient of stem-ferritin nanoparticles across SMARTA, BL/6 and BALB/c mice; the broad ferritin-specific CD4+ T cell pool in BALB/c mice was associated with 10-fold higher stem antibody titres compared to BL/6 animals. This appears to be generally consistent in genetically diverse human cohorts as well: large glycoproteins such as SARS-CoV-2 spike contain sufficient epitopes restricted by a broad array of Human Leucocyte Antigen (HLA) alleles to support immunogenicity in diverse populations. Small antigens like HA stem, SARS-CoV-2 RBD, HIV env or Plasmodium circumsporozoite protein (CSP), however, contain reduced numbers of CD4+ T cell epitopes that begin to restrict the quantity of help available in certain individuals or populations.^15^, ^16^, ^17^, ^18^ At this point, vaccine immunogens are at higher risk of failure, particularly for pathogens with high antigenic diversity.^16^

Considering these factors, it becomes essential to understand the minimal requirements for CD4+ T cell help to support a robust GC reaction. Our data identify distinct scenarios under which vaccination can succeed or fail to generate a serological response. In the context of multimeric arrayed antigens, highly subdominant T cell help becomes sufficient to drive a vaccine-specific serological response. Despite using multiple assays, the frequency of stem- or ferritin-specific CD4+ T cells elicited by primary vaccination of BL6 mice appears to be below our limit of detection. This is consistent with prior work by Nelson et al., who identified robust ferritin-specific CD4 T cell responses only in BALB/c, but not BL/6, mice.^21^ Nonetheless, the lack of stem-ferritin immunogenicity in SMARTA transgenic mice and ferritin-specific proliferative responses evident upon repeated boosting clearly support the contribution of low frequency or low affinity antigen-specific T cells to vaccine immunogenicity. Mechanistically, the augmented BCR crosslinking and NF-kB signalling induced by nanoparticle vaccines^20^ may potentially reduce the magnitude and/or quality of CD4+ T cell help required to initiate a stem-specific GC B cell response.

In the context of soluble non-arrayed antigens, we find that a single CD4+ T cell epitope is sufficient to support an antigen-specific GC reaction, but perhaps more importantly, find that only some epitopes drive CD4+ T cell responses capable of providing suitable B cell help. The potent immunogenicity of the stem-GP_61_ protein clearly demonstrates how even a comparatively restricted CD4+ T cell repertoire can support robust GC activity, as the naive repertoire of GP_61_-specific T cells is heavily biased toward TRAV14/TRAV14D and TRBV13/TRBV31 gene usage.^30^ Our data therefore suggest that a highly diverse TFH pool may not be required for vaccine efficacy; rather, the rational selection of a small number of high-quality class II epitopes could be sufficient to support robust humoural immunity.

Collectively, our data extend the two-step model^29^^,^^31^ of epitope-specific GC TFH development, in which naïve T cell priming by DCs which drives immunodominance and pre-TFH phenotype but not GC TFH selection, while subsequent T cell-B cell interactions down select T cell specificities to populate the GC TFH pool. During the first 5 days post-vaccination, the kinetics of T cell expansion were comparable between HA_91_ and GP_61_ epitopes, likely reflecting naïve T cell precursor frequencies which are known to be high for GP_61_ compared to other epitopes such as OTII.^30^^,^^32^ Interestingly, early DC priming was markedly efficient, as all doses of stem-GP61 antigen tested (0.25 μg to 5 μg) were saturating for T cell effector frequency, even when they compromised TFH numbers. Comparable observations have been reported for transgenic OTII cells (at doses of 10 μg to 100 μg),^33^ suggesting similar dose-dependent regulation of TFH differentiation for monoclonal vs polyclonal and low affinity vs high affinity TCRs. Indeed, both quantity and duration of antigen availability have emerged as key determinants of TFH differentiation and GC longevity in multiple systems.^29^^,^^34^ It is currently unclear whether this is driven by differential antigen processing and presentation in B cells versus DCs, more efficient acquisition by DCs due to immunogen draining patterns in the LN, or a selective threshold imposed by low-frequency CD4+ T cell/B cell interactions at the T:B border.

It is well known that the major selective event for TFH differentiation and GC residence is recognition of pMHCII complexes presented by B cells.^33^ However, our data suggest that even in a polyclonal context, there are stark differences in the capacity of epitope-specific T cell populations to provide B cell help. Mechanistically, this appears to be governed by a low propensity of some polyclonal populations (like HA_91_-reactive T cells) to undergo differentiation into CD90lo GC-resident TFH. Our panel of stem-peptide immunogens identified some ‘immunocapable’ epitopes (GP_61_ and HA_523_), which support GC responses using the endogenous T cell and B cell repertoire in BL/6 mice. Other epitopes, such as HA_91_, required manipulation of B:T interactions (e.g. expansion of the peptide-presenting B cell pool) to effectively drive GC. The rescue of stem IgG by co-immunisation of stem-HA_91_ and OVA-HA_91_ is consistent with a positive feedback loop of signalling between TFH and GC B cells. OVA-specific B cells receive “competent” T cell help from multiple OVA-derived class II epitopes, promoting their activation, proliferation and maturation. We speculate that subsequent presentation of HA_91_/IA^b^ by now activated B cells can, in this context, support the differentiation and GC recruitment of HA_91_-specific T cells, positioning them to provide help to stem-specific B cells. Possible mechanisms underpinning the divergent ‘helpfulness’ of different epitopes include peptide-intrinsic differences in presentation/processing by B cells (but not DCs) or epitope-specific differences in TCR/MHCII avidity at an aggregate, polyclonal level. The linear relationship between dose of stem-GP_61_ and GP_61_ TFH differentiation suggest the absolute densities of IAb/GP_61_ on the surface of stem-specific B cells dictates a propensity to recruit GP_61_ T cells into the GC, potentially favouring a deterministic role for B cell peptide presentation in controlling TFH composition.

This study has several limitations that may be addressed in future research. While we demonstrated the impact of CD4 help on antigen-specific GC B cells, we were unable to address whether such help impacted the antigenic space explored by the serological response. Further characterisation of antibody neutralising activity and protection in infectious challenge models will be valuable. In addition, future work should address the mechanisms underpinning the observed differences in Tfh differentiation between immunodominant peptides, and assess the generalisability of these findings to a wider array of vaccine platforms and antigens.

Additional study is required to extend these observations into human cohorts. Despite the incredible diversity provided by HLA polymorphism and TCR repertoires, mounting evidence suggests that the human TFH repertoire may also be relatively restricted, with individual epitopes or class II alleles exerting substantial impacts on the outcome of vaccination.^22^^,^^35^ Antigens such as HIV env, *Plasmodium* CSP and SARS-CoV-2 RBD frequently contain only 1–2 epitopes that are recognised on an individual level.^15^, ^16^, ^17^, ^18^ The qualitative differences between HA_91_ and GP_61_ highlight the potential pitfalls of relying on few, endogenous epitopes for consistent vaccine immunogenicity. Further work is required to clarify whether multiple, subdominant epitopes provide additive or synergistic support for GC B cell development. These data also underscore the need to accurately predict the quality of B cell help, rather than just immunogenicity or immunodominance, of any given epitope and responding T cell population to aid vaccine design efforts.

## Contributors

Conceptualisation: JAJ, AKW, HXT, MZMZ, CAG; Data curation: MZMZ, JAJ; Formal analysis: MZMZ, HXT, JAJ; Funding acquisition: JAJ, AKW, HXT; Investigation—Animal handling and vaccinations: MZMZ, HXT, LM; Investigation—*in vitro* stimulation assays and ELISAs: KMW, DP, HXT; Investigation—flow cytometry and antigen-specific T/B cell kinetics: MZMZ, KMW; Resources: AK, RE, CAG; Supervision: JAJ, AKW, HXT; Writing—original draft: JAJ, AKW; Writing—review and editing: JAJ, AKW, MZMZ, HXT; Validation: JAJ, MZMZ and KMW have accessed and verified the underlying data in the manuscript. All authors have read and approved the final version of the manuscript.

## Data sharing statement

All data are presented in the manuscript and Supplementary Files. Raw data are available from the corresponding author upon reasonable request.

## Declaration of interests

The authors declare no competing interests.

## References

[1] Wrapp D., Wang N., Corbett K.S. (2020). Cryo-EM structure of the 2019-nCoV spike in the prefusion conformation. Science.

[2] Corbett K.S., Edwards D.K., Leist S.R. (2020). SARS-CoV-2 mRNA vaccine design enabled by prototype pathogen preparedness. Nature.

[3] Tian J.H., Patel N., Haupt R. (2021). SARS-CoV-2 spike glycoprotein vaccine candidate NVX-CoV2373 immunogenicity in baboons and protection in mice. Nat Commun.

[4] Bos R., Rutten L., van der Lubbe J.E.M. (2020). Ad26 vector-based COVID-19 vaccine encoding a prefusion-stabilized SARS-CoV-2 Spike immunogen induces potent humoral and cellular immune responses. NPJ Vaccines.

[5] Crank M.C., Ruckwardt T.J., Chen M. (2019). A proof of concept for structure-based vaccine design targeting RSV in humans. Science.

[6] Miranda M.C., Kepl E., Navarro M.J. (2024). Potent neutralization of SARS-CoV-2 variants by RBD nanoparticle and prefusion-stabilized spike immunogens. NPJ Vaccines.

[7] Nolan T.M., Deliyannis G., Griffith M. (2023). Interim results from a phase I randomized, placebo-controlled trial of novel SARS-CoV-2 beta variant receptor-binding domain recombinant protein and mRNA vaccines as a 4th dose booster. EBioMedicine.

[8] Widge A.T., Hofstetter A.R., Houser K.V. (2023). An influenza hemagglutinin stem nanoparticle vaccine induces cross-group 1 neutralizing antibodies in healthy adults. Sci Transl Med.

[9] Tan H.X., Jegaskanda S., Juno J.A. (2019). Subdominance and poor intrinsic immunogenicity limit humoral immunity targeting influenza HA stem. J Clin Invest.

[10] Tan H.X., Juno J.A., Lee W.S. (2021). Immunogenicity of prime-boost protein subunit vaccine strategies against SARS-CoV-2 in mice and macaques. Nat Commun.

[11] Houser K.V., Gaudinski M.R., Happe M. (2022). Safety and immunogenicity of an HIV-1 prefusion-stabilized envelope trimer (Trimer 4571) vaccine in healthy adults: a first-in-human open-label, randomized, dose-escalation, phase 1 clinical trial. EClinicalMedicine.

[12] Hernández-Bernal F., Ricardo-Cobas M.C., Martín-Bauta Y. (2022). Safety, tolerability, and immunogenicity of a SARS-CoV-2 recombinant spike RBD protein vaccine: a randomised, double-blind, placebo-controlled, phase 1-2 clinical trial (ABDALA Study). EClinicalMedicine.

[13] Lee J.H., Hu J.K., Georgeson E. (2021). Modulating the quantity of HIV Env-specific CD4 T cell help promotes rare B cell responses in germinal centers. J Exp Med.

[14] Sarkar S., Kalia V., Murphey-Corb M., Montelaro R.C. (2002). Detailed analysis of CD4+ Th responses to envelope and Gag proteins of simian immunodeficiency virus reveals an exclusion of broadly reactive Th epitopes from the glycosylated regions of envelope. J Immunol.

[15] Grifoni A., Sidney J., Vita R. (2021). SARS-CoV-2 human T cell epitopes: adaptive immune response against COVID-19. Cell Host Microbe.

[16] Wahl I., Obraztsova A.S., Puchan J. (2022). Clonal evolution and TCR specificity of the human T(FH) cell response to Plasmodium falciparum CSP. Sci Immunol.

[17] Cohen K.W., Fiore-Gartland A., Walsh S.R. (2023). Trivalent mosaic or consensus HIV immunogens prime humoral and broader cellular immune responses in adults. J Clin Invest.

[18] Juno J.A., Tan H.X., Lee W.S. (2020). Humoral and circulating follicular helper T cell responses in recovered patients with COVID-19. Nat Med.

[19] Grewal I.S., Moudgil K.D., Sercarz E.E. (1995). Hindrance of binding to class II major histocompatibility complex molecules by a single amino acid residue contiguous to a determinant leads to crypticity of the determinant as well as lack of response to the protein antigen. Proc Natl Acad Sci U S A.

[20] Brooks J.F., Riggs J., Mueller J.L. (2023). Molecular basis for potent B cell responses to antigen displayed on particles of viral size. Nat Immunol.

[21] Nelson S.A., Richards K.A., Glover M.A. (2022). CD4 T cell epitope abundance in ferritin core potentiates responses to hemagglutinin nanoparticle vaccines. NPJ Vaccines.

[22] Mallajosyula V., Chakraborty S., Sola E. (2024). Coupling antigens from multiple subtypes of influenza can broaden antibody and T cell responses. Science.

[23] Ada G., Isaacs D. (2003). Carbohydrate-protein conjugate vaccines. Clin Microbiol Infect.

[24] Kelly H.G., Tan H.X., Juno J.A. (2020). Self-assembling influenza nanoparticle vaccines drive extended germinal center activity and memory B cell maturation. JCI Insight.

[25] Yassine H.M., Boyington J.C., McTamney P.M. (2015). Hemagglutinin-stem nanoparticles generate heterosubtypic influenza protection. Nat Med.

[26] Schindelin J., Arganda-Carreras I., Frise E. (2012). Fiji: an open-source platform for biological-image analysis. Nat Methods.

[27] Moin S.M., Boyington J.C., Boyoglu-Barnum S. (2022). Co-immunization with hemagglutinin stem immunogens elicits cross-group neutralizing antibodies and broad protection against influenza A viruses. Immunity.

[28] Yeh C.H., Finney J., Okada T., Kurosaki T., Kelsoe G. (2022). Primary germinal center-resident T follicular helper cells are a physiologically distinct subset of CXCR5(hi)PD-1(hi) T follicular helper cells. Immunity.

[29] Deenick E.K., Chan A., Ma C.S. (2010). Follicular helper T cell differentiation requires continuous antigen presentation that is independent of unique B cell signaling. Immunity.

[30] Khatun A., Kasmani M.Y., Zander R. (2021). Single-cell lineage mapping of a diverse virus-specific naive CD4 T cell repertoire. J Exp Med.

[31] Goenka R., Barnett L.G., Silver J.S. (2011). Cutting edge: dendritic cell-restricted antigen presentation initiates the follicular helper T cell program but cannot complete ultimate effector differentiation. J Immunol.

[32] Moon J.J., Chu H.H., Pepper M. (2007). Naive CD4(+) T cell frequency varies for different epitopes and predicts repertoire diversity and response magnitude. Immunity.

[33] Baumjohann D., Preite S., Reboldi A. (2013). Persistent antigen and germinal center B cells sustain T follicular helper cell responses and phenotype. Immunity.

[34] Tam H.H., Melo M.B., Kang M. (2016). Sustained antigen availability during germinal center initiation enhances antibody responses to vaccination. Proc Natl Acad Sci U S A.

[35] Mentzer A.J., Dilthey A.T., Pollard M. (2024). High-resolution African HLA resource uncovers HLA-DRB1 expression effects underlying vaccine response. Nat Med.

