# Supplementary material for: Immunodominance is a poor predictor of vaccine-induced T follicular helper cell quality
Source: eBioMedicine. 2026 Feb 26;125:106185. doi: 10.1016/j.ebiom.2026.106185 (PMC12962106; doi:10.1016/j.ebiom.2026.106185)
Supplement: Supplementary Figures [file mmc1.pdf]

Supplementary Figures

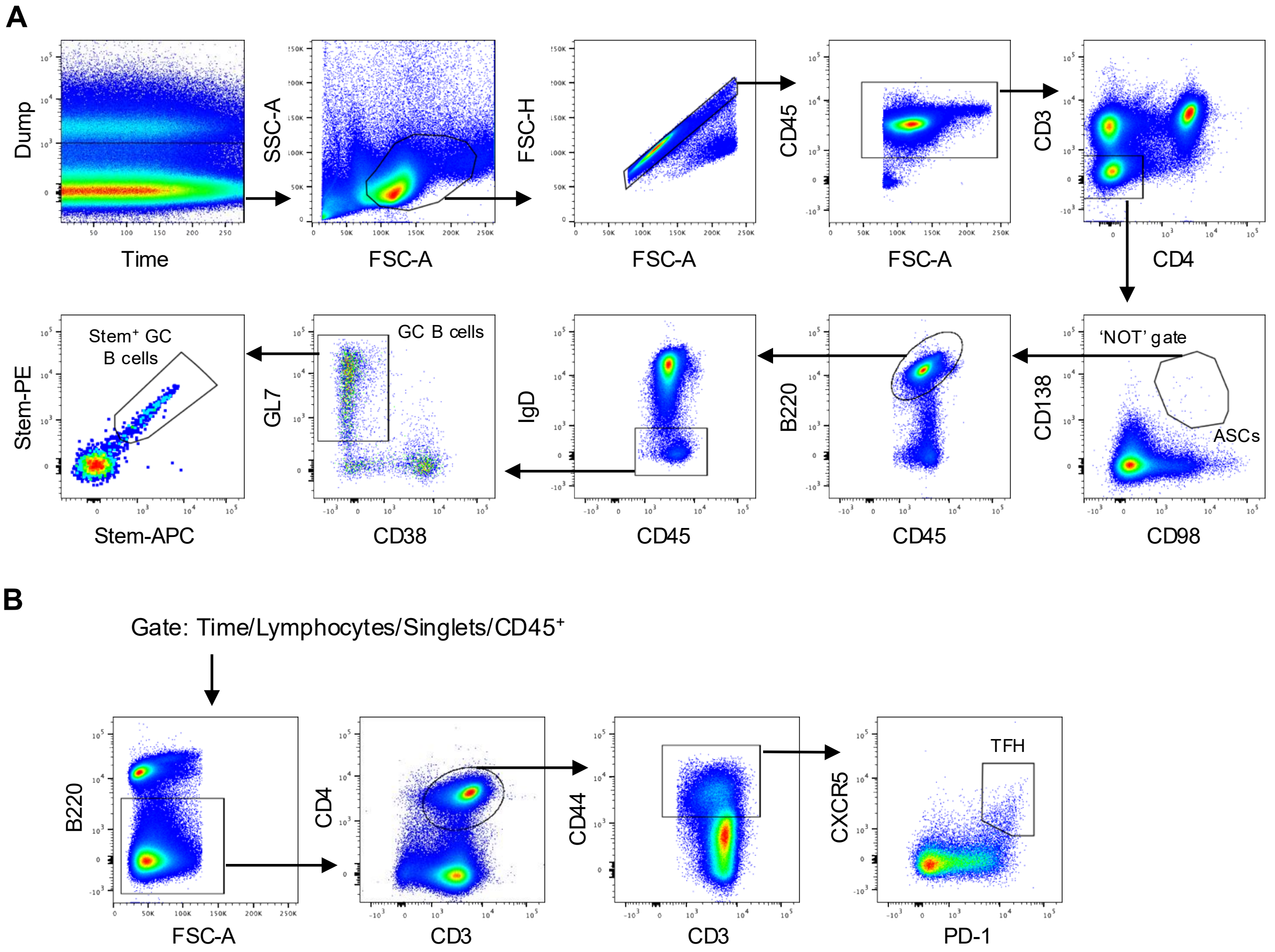

Supplementary Figure 1. Gating strategy for GC B cell and TFH populations.
(A) Lymphocyte populations are gated through a Dump (F4/80 and live/dead) vs Time plot, then identified by forward and side scatter, with exclusion of doublets (FSC-A vs FSC-H) and selection of CD45^{+} cells. Antibody secreting cells are defined as CD3^{-}CD4^{-} cells that co-express CD138 and CD98. Class-switched B cells are defined as non-ASCs that are B220^{+} and IgD^{-}. Germinal centre B cells are gated as GL7^{+}CD38^{lo}, and antigen specificity determined by staining with recombinant protein tetramers. **(B)** CD4^{+} T cells are identified as B220^{-} and CD3^{+}CD4^{+}. Antigen-experienced cells are gated as CD44^{hi}, and TFH are defined as CXCR5^{+}PD-1^{+} cells.

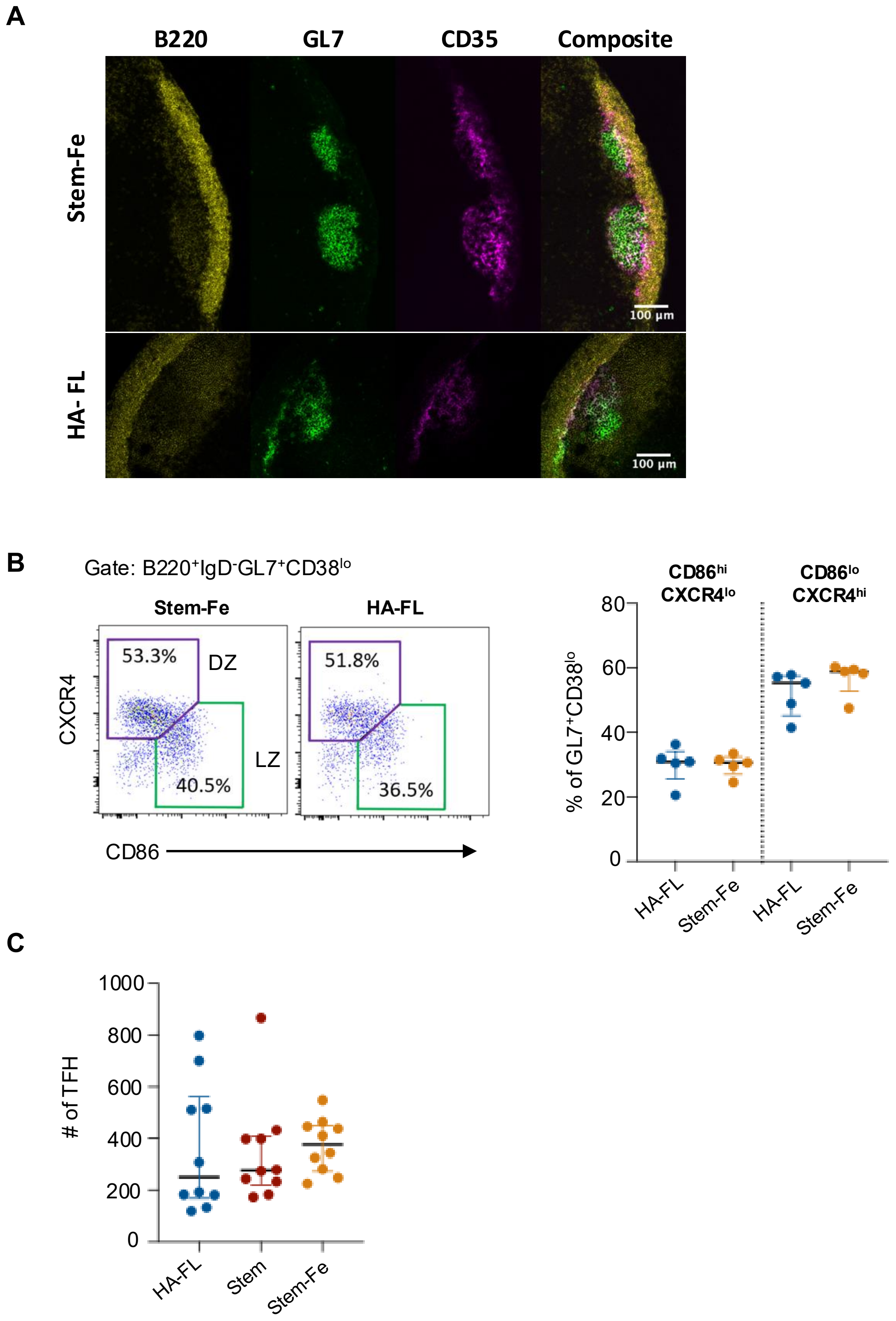

Supplementary Figure 2. Germinal centre features following stem-Fe vaccination. **(A)** Confocal imaging of vaccine draining lymph nodes showing localization of B220^{+} B cells (yellow), GL7^{+} GC B cells (green), and CD35^{+} follicular dendritic cells (magenta). **(B)** Identification of light zone (LZ; CXCR4^{lo}CD86^{hi}) and dark zone (DZ; CXCR4^{hi}CD86^{lo}) GC B cells in stem-Fe (top) or HA-FL (bottom) vaccinated animals (N=5/group). **(C)** Number of CXCR5^{hi}PD-1^{hi} TFH in the draining LN at day 14 following HA-FL, HA-stem, or stem-ferritin nanoparticles (N=10/group). Lines indicate median and IQR.

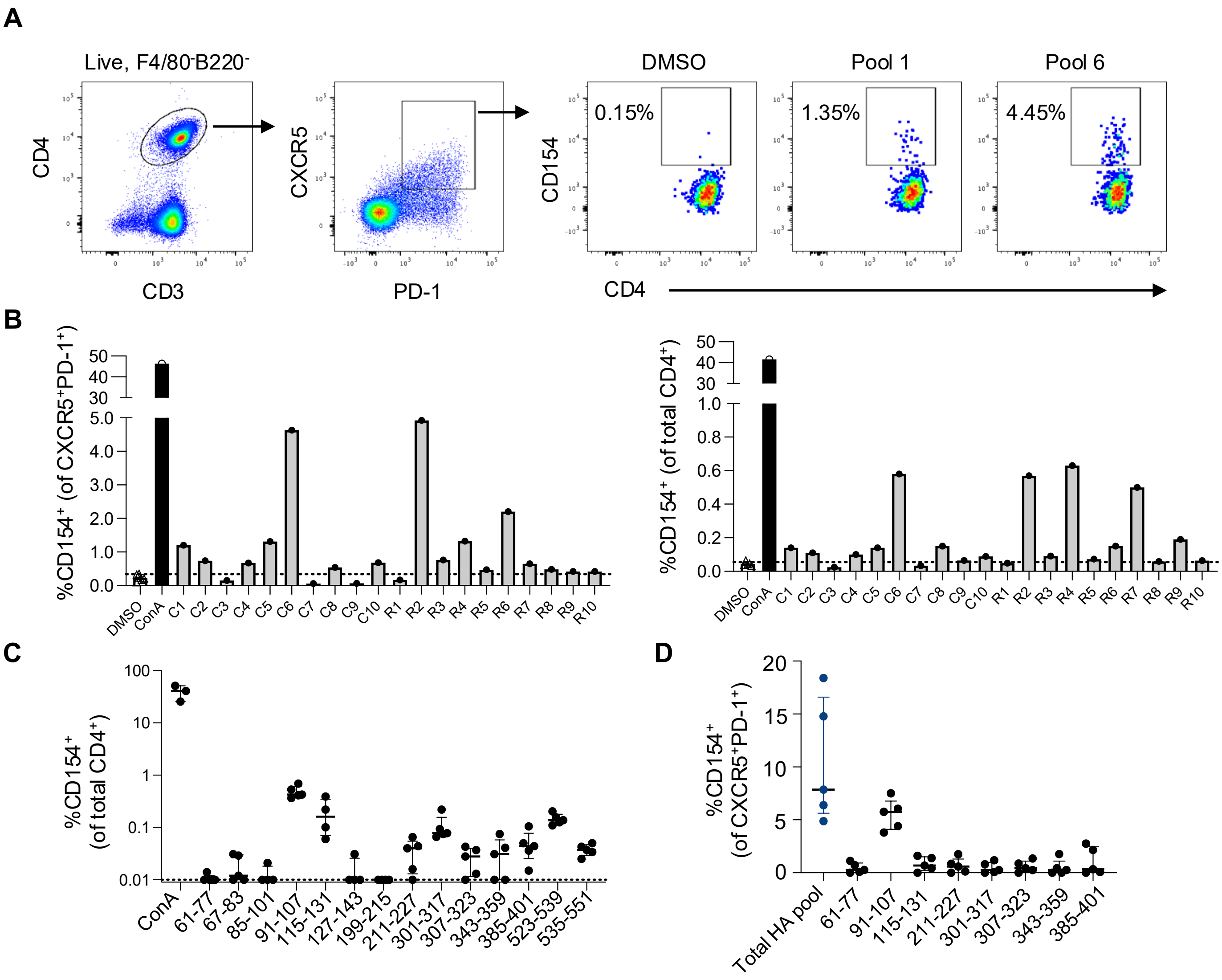

Supplementary Figure 3. Identification of immunogenic peptides in PR8 HA. (A) Representative staining of CD154 upregulation on CXCR5⁺PD-1⁺ CD4⁺ T cells in response to *in vitro* stimulation with peptide pools covering the PR8 HA protein. **(B)** Frequencies of peptide-specific or ConA-responsive cells among CXCR5⁺ (left) or total (right) CD4⁺ T cells following PR8 infection. Each data point represents a single replicate using pooled lymph node samples from multiple mice. Dashed line indicates maximum value of DMSO control replicates. **(C)** Frequencies of peptide-specific bulk CD4⁺ T cell responses in the mediastinal LN at day 14 post-PR8 infection. Each dot represents a single mouse (n=4-5 per group). Dashed line indicates limit of detection (0.01%). **(D)** Peptide- or pool-specific TFH responses in draining LN at day 14 following intramuscular vaccination with soluble PR8 HA antigen.

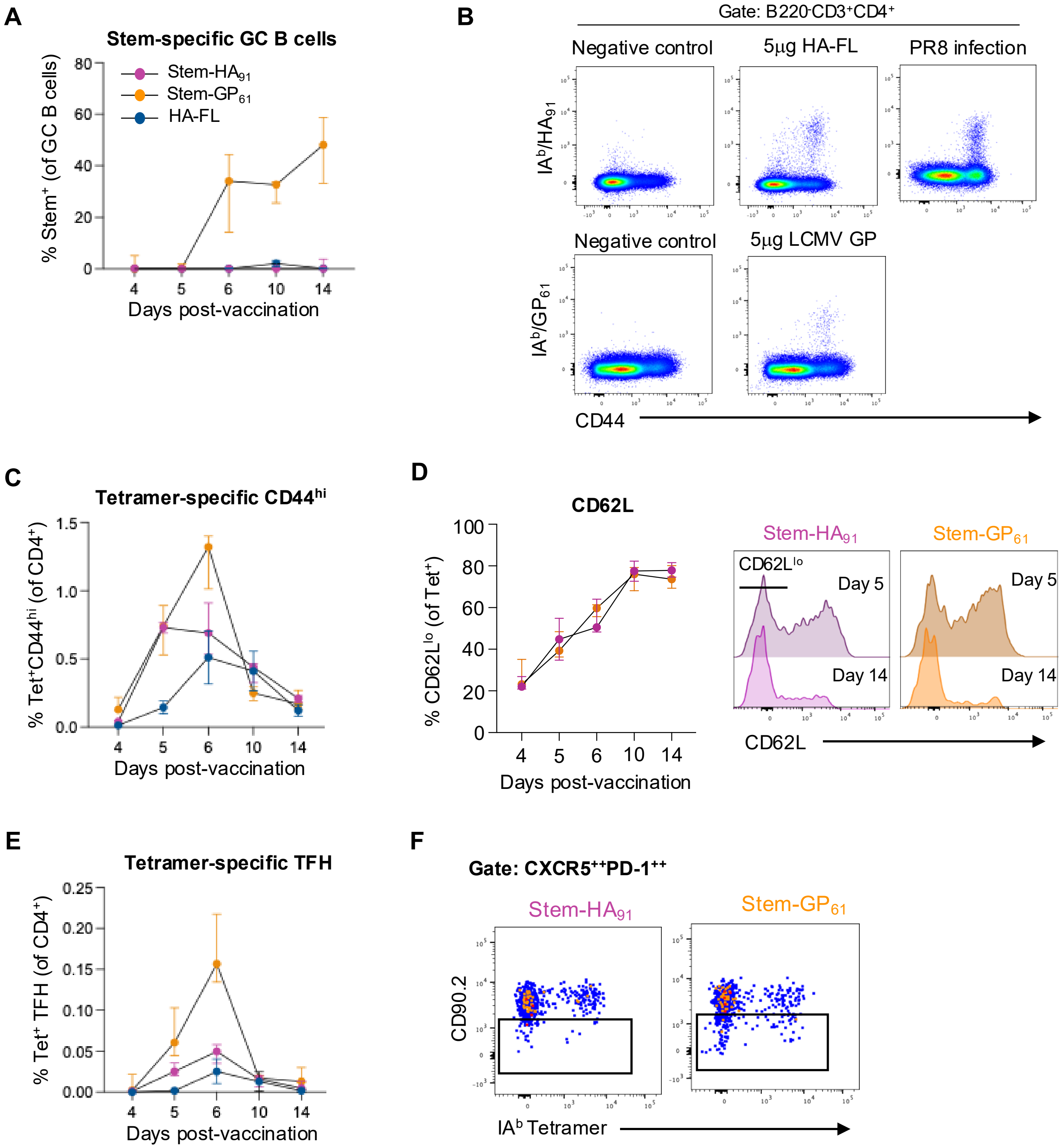

Supplementary Figure 4. Detection of antigen-specific CD4+ T cells following vaccination or infection. (A) Longitudinal tracking of stem-specific GC B cells in the draining LN at days 4, 5, 6, 10 and 14 post-vaccination with HA-FL, stem-HA_{91} or stem-GP_{61} (N=5/group). **(B)** Representative staining of IA^b/HA_{91} and IA^b/GP_{61} tetramers in draining lymph nodes at day 14 following HA-FL vaccination, PR8 infection or LCMV GP vaccination. **(C)** Frequency of and **(D)** CD62L expression on tetramer-specific memory cells (CD44hi) from days 4-14 post-vaccination (N=5 per group). **(E)** Frequency of tetramer-specific TFH ($CXCR5^{hi}PD\text{-}1^{hi}$) at days 4, 5, 6, 10 and 14 post-vaccination. **(F)** Representative plots showing CD90 expression on tetramer+ and tetramer- TFH populations in the dLN at day 10 post-vaccination. Symbols indicate median and IQR (n=5 per group).
